# Barriers to Low-Vision Rehabilitation Services for Visually Impaired Patients in a Multidisciplinary Ophthalmology Outpatient Practice

**DOI:** 10.1155/2021/6122246

**Published:** 2021-11-29

**Authors:** Karima S. Khimani, Carissa R. Battle, Lauren Malaya, Aaleena Zaidi, Mary Schmitz-Brown, Huey-Ming Tzeng, Praveena K. Gupta

**Affiliations:** ^1^Department of Ophthalmology and Visual Sciences, University of Texas Medical Branch, Galveston, TX 77555, USA; ^2^School of Medicine, University of Texas Medical Branch, Galveston, TX 77555, USA; ^3^School of Nursing, University of Texas Medical Branch, Galveston, TX 77555, USA

## Abstract

Low-vision rehabilitation (LVR) has significant benefit in improving the quality of life of visually impaired patients. However, these services are highly underutilized in ophthalmology practices. A quality improvement study was performed to investigate barriers to LVR services for patients at the University of Texas Medical Branch (UTMB) between 2010 and 2020. Low vision was defined as the best corrected visual acuity of 20/70 or worse in the better-seeing eye or a visual field less than 20 degrees. Potential subjects were screened (*n* = 577) from the electronic medical record using International Classification of Disease (ICD) codes for legal blindness, impaired vision, and low vision. Chart review identified 190 subjects who met criteria for low-vision analysis. Patients who received LVR referrals to attend at least one LVR service visit from the eligible subjects were contacted for participation in phone interviews regarding their LVR experience. Practicing eye care providers (ECPs) at UTMB completed a questionnaire to capture their referral patterns. Of the eligible subjects, 64% were referred to LVR services by ECPs. Reported patient barriers included mental health issues (76%), denial of need for low-vision aid (71%), poor physical health (67%), lack of transportation (57.1%), and lack of referrals (36%). EPCs reported patient's overall health (67%), older age (44%), lack of social support (44%), poor cognitive function (44%), and low likelihood of follow-up (44%) as barriers to referring patients to LVR. This study identified several modifiable barriers that can be addressed to access LVR services for low-vision patients. Changing referral patterns, eliminating variations in referral criteria, and increasing patient awareness and knowledge of LVR resources may tremendously improve the quality of life of low-vision patients.

## 1. Introduction

Low vision has a significant global burden, affecting 2.2 billion people worldwide and 2.9 million people aged 40 years and above in the United States [1, 2]. Low vision is defined as a visual acuity of 20/70 or less in the better-seeing eye. However, this numeric value does not capture the functional challenges of low-vision patients in performing their daily activities. Therefore, the National Eye Institute (NEI) adopted a newer definition of low vision that includes visual impairment with functionally disabling factors such as vision loss impairing learning, vocational or avocational pursuits, social interaction, or the activities of daily living [3]. Almost every patient with low vision experiences some form of difficulty in completing vision-related daily tasks, which can lead to diminished quality of life and substantial social impacts. As the average lifespan of the population continues to increase, the prevalence of individuals with low vision will undoubtedly trend upward because vision impairment disproportionately affects the elderly. Adults over the age of 80 account for almost 70% of individuals with severe vision impairment (visual acuity 20/200 or less in the better eye) [4].

The leading causes of low vision are age-related macular degeneration, diabetic retinopathy, glaucoma, retinitis pigmentosa, and adult-onset foveomacular vitelliform dystrophy (AOFVD) [5, 6]. Age-related macular degeneration is a progressive, chronic disease that leads to deterioration of central vision, which can complicate many daily living activities for affected patients [7, 8]. AOFVD is another form of macular degeneration, characterized as a heterogenous group of disorders that causes progressive central scotoma and foveal damage, requiring patients to adopt self-adaptive strategies to compensate for daily activities like reading [6]. On the other hand, glaucoma causes peripheral vision loss in its advanced stages, causing visual field deficits of 20 degrees or less [9]. Diabetes is a leading worldwide public health concern and can lead to a complex array of microvascular and neuronal complications, including diabetic retinopathy, which is the leading cause of low vision in the United States and increasing cause of visual impairment globally [10]. The most common genetic cause of low vision is retinitis pigmentosa, a degenerative disease that begins with night blindness and progresses with gradual peripheral vision loss [11]. Low-vision rehabilitation (LVR) is a valuable option for many patients coping with vision loss related to these conditions, especially when medical or surgical interventions are either contraindicated or unsuccessful.

LVR services have been shown to significantly benefit individuals with low vision in improving their daily living activities [12]. These services can be provided by licensed specialists in ophthalmology, optometry, or low-vision-specialized occupational therapists. This care involves a dynamic, personalized, physician-patient approach to enhance the patient's vision and cater to individual vision-related goals. Patients have a variety of treatment modalities to choose from depending on their needs and comfort. Examples of low-vision aids include vision rehabilitation training, standard and electronic modalities (i.e., reading enhancers, magnifiers, color vision enhancers, and solar shields), and surgical options (i.e., retinal prostheses) [13]. These rehabilitation and training techniques can help patients drive, improve their mobility, assist with facial recognition, help with reading and writing, enhance their color vision, and alleviate emotional distress. Previous research has shown that patients are not familiar with the types of services and aids that LVR provides [14]. Therefore, patients must rely heavily on their primary eye care providers (ECPs) for descriptions of the benefits of LVR services.

Providing LVR services is beyond the scope of most ECPs, as their primary focus often is geared towards treating the underlying cause of low vision. However, it is highly recommended by the American Academy of Ophthalmology (AAO) that the treating ECP take the initiative to simultaneously refer a low-vision patient to LVR services for timely help, especially if the nature of the disease is progressive [15]. Often, ophthalmologists refer their low-vision patients in two scenarios: (1) they have depleted all other avenues of services or (2) the patient is only able to either count fingers or see hand motions [16]. Thus, the importance of early and timely referral to LVR services cannot be overlooked.

Although multiple practice guidelines have emphasized the importance of referral to LVR [13], only 5–10% of patients who qualify for LVR services end up obtaining them [17]. In a review study, Luu et al. [18] identified potential barriers to accessing LVR services and proposed a holistic model of low-vision care for improving vision-related quality of life. Barriers identified in this study included lack of appropriate referrals by optometrists and ophthalmologists due to unawareness of available services and lack of patient understanding about the referrals. Reported barriers also included health negligence by the patient due to a variety of personal and social factors, including depression, denial of need for low-vision aid, presence of social stigma, transportation issues, and perceived costs of vision services [18, 19]. Underutilization and inaccessibility of LVR services have been identified in both developed and developing countries, including the United States [18]. The Montreal Barriers Study found that of the 702 patients who qualified as low vision, 54% used LVR services, 33% were never referred and/or were unfamiliar with LVR services, and 13% were aware of LVR services but elected not to use them [16]. Thus, there is a need to facilitate LVR services between ECPs and patients by addressing the gaps in the referral system.

Our study aimed to identify barriers encountered by low-vision patients that affect their opportunity and ability to receive LVR services from ophthalmology/optometry providers in a hospital setting at the University of Texas Medical Branch (UTMB) in Galveston, Texas. To our best knowledge, this is the first quality improvement study conducted in the United States that addresses the barriers to access LVR services from a patient and clinician perspective. We collected data from three resources to achieve our study purpose: retrospective chart reviews, phone interviews with low-vision patients or their caregivers, and an online survey to ECPs practicing ophthalmology or optometry in the same center. The data collected will help in establishing a stratified system in continuum of care from ECP to LVR services without compromising potential outcomes.

## 2. Materials and Methods

This retrospective study was approved by the local institutional review board of the University of Texas Medical Branch (approval number 20-0112) and was conducted in accordance with Good Clinical Practice and the tenets of Helsinki Declaration.

**Figure 1:**
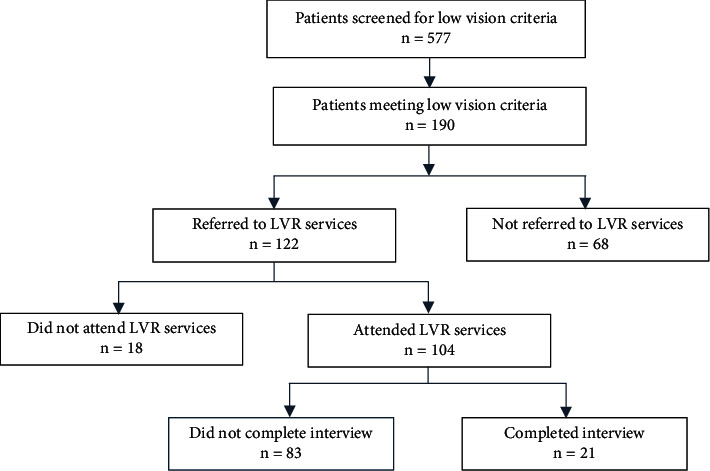
Eligible low-vision patients screened, referred, and interviewed. LVR = low-vision rehabilitation.

A retrospective chart review was performed to identify low-vision patients seen by ECPs at the University of Texas Medical Branch from year 2010 to 2020. Low vision was defined as having a best corrected visual acuity of 20/70 or worse in the better eye, or a visual field of less than 20 degrees, and not correctable by medical, surgical, or refractive interventions. Potential subjects were screened (*n* = 577) from the EPIC electronic medical record (EMR) database using the International Classification of Disease (ICD) codes for legal blindness, impaired vision, and low vision. On further chart review, 190 subjects met eligibility criteria for low-vision study analysis. Age, gender, and etiology of low vision were also collected from the EMR. Patients given LVR referrals were identified, and the research team further narrowed the study population to those who attended at least one LVR service visit (Figure 1).

**Figure 2:**
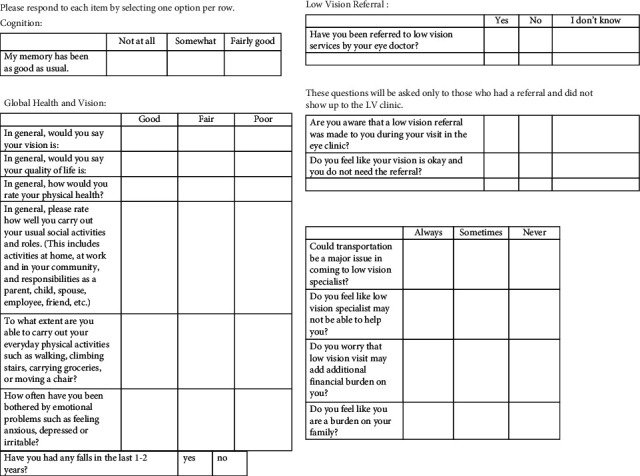
Low-vision patient questionnaire. Patients provided responses during phone interviews with members of the research team.

The qualitative part of this study involved capturing the referral experiences of patients and ECPs. Patients with LVR referrals were contacted for participation in phone interviews after verbal consent was obtained. Patients were excluded from the study if they were less than 18 years of age, were deceased, had severe cognitive impairment or psychiatric disorders, did not speak English, or were seen at UTMB for a second opinion only. Twenty-one low-vision patients agreed to take the questionnaire (Figure 2). Patients responded to questions regarding their health status, mentation, cognition, perception of vision, and referral experience on a graded scale as shown in Figure 2. Responses were documented by the interviewers on forms that were stored in a secure database.

Another questionnaire was administered to the practicing ECPs at UTMB who were invited to participate in an online survey. Participation was anonymous, and consent was obtained prior to initiation of the survey. A total of 9 ECPs successfully completed the survey. The participating ECPs gave information regarding their years of practice, familiarity with low-vision aids, use of Preferred Practice Patterns guidelines, number of low-vision patients seen and referred per month, and perceived barriers to low-vision referrals. Responses to patient and provider questionnaires were analyzed and tabulated in percentages for analysis.

## 3. Results and Discussion

### 3.1. Patient Population

Of the 190 eligible low-vision patients, 57% were females and had a mean age of 72.5 (range 51–94). The most common etiologies of low vision included age-related macular degeneration (35%), diabetic retinopathy (16%), and glaucoma (16%). Less common etiologies included optic neuropathy (5%), retinal detachment (3%), and chronic uveitis (2%) (see Table 1). Nine ECPs were included in the study, of which 7 had at least 10 years of practice in the field of ophthalmology. All ECPs were board-certified, trained in the United States, and worked at the UTMB Department of Ophthalmology and Visual Sciences.

### 3.2. Perceived Barriers to LVR Service Referrals by ECPs

**Figure 3:**
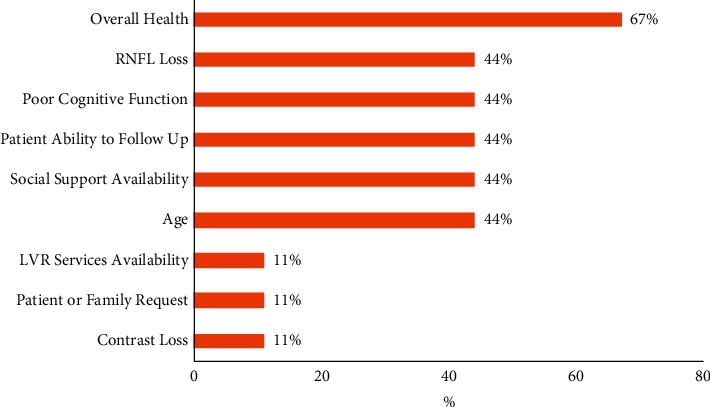
Barriers to low-vision rehabilitation services from online survey evaluating eye care provider referral practices. LVR = low-vision rehabilitation; RNFL = retinal nerve fiber layer.

Of the 9 ECPs who completed the online survey, 78% of ECPs reported being familiar with low-vision services and 89% reported using Preferred Practice Pattern guidelines recommended by AAO. Most ECPs (89%) reported referring eligible patients to LVR services in their practices when appropriate. Responses from the ECP questionnaire were analyzed, and commonly perceived barriers to LVR referrals were identified (Figure 3). The most reported factors for not referring low-vision patients to LVR services included patient's overall health (67%), older age (44%), lack of social support (44%), poor cognitive function (44%), and less likelihood of following up with LVR services (44%). In addition, a few ECPs reported lack of LVR availability (11%), lack of patient or family request for LVR (11%), and contrast loss (11%) as barriers to referral.

### 3.3. Perceived Barriers to LVR Services for Low-Vision Patients

**Figure 4:**
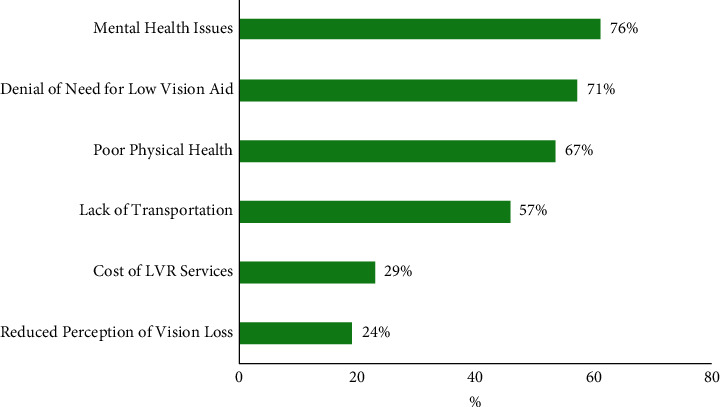
Barriers to low-vision rehabilitation services from the questionnaire evaluating patient experiences. LVR = low-vision rehabilitation.

Of the 190 patients identified as meeting low-vision criteria, 64% were found to have LVR referrals. Only 55% (104/190) attended LVR services (Figure 1). Of the 104 patients who attended at least one LVR service appointment, 21 completed phone interviews regarding their referral experience. Qualitative analysis of the questionnaire responses revealed the following barriers to utilizing LVR services: mental health issues including anxiety and depression (76%), denial of need for low-vision aid (71%), poor physical health (67%), lack of transportation (57%), and lack of referrals (36%) (Figure 4). Communication failure was also identified, as 50% of patients did not know whether they had been referred to LVR.

### 3.4. Discussion

This study used three data sources (i.e., chart reviews, patient phone interviews, and ECPeye surveys) to identify the most common barriers to LVR services at a tertiary outpatient eye center. The referral completion rate at our institution was in line with similar studies [16]. At UTMB eye care, 55% of low-vision patients utilized LVR services and 9% of the patients who completed phone interviews were either not aware of their referral or chose not to schedule an LVR appointment.

Specific barriers impeding the completion of LVR referrals have been addressed in many studies [20–27]. A 2017 study by Matti et al. reported that 27% of patients declined LVR services due to poor health, another 27% declined as they felt LVR was not necessary, and 10% did not feel that LVR services would be beneficial [25]. In our study, 67% of patients cited poor health and 71% denied the necessity of LVR services.

Depression and anxiety are common in the elderly population and seem to increase in patients with low vision. In this study, 76% of patients reported mental health issues, which may have contributed to underutilization of LVR services. A recent cross-sectional study at a publicly funded, comprehensive eye clinic in Alabama found high average scores on standardized screening for depression in its low-vision population [14]. Furthermore, the social stigma associated with using low-vision aids may further prevent patients from utilizing LVR services.

More than half of the patients who completed the phone interview questionnaire reported lack of transportation as a barrier to LVR participation. This is consistent with other studies which found transportation challenges as a common barrier to accessing services [14, 16, 27, 28]. Less than one-third of the patients perceived the cost of LVR services as a barrier, a barrier relatively unique to the population of the United States and therefore addressed in a few studies [20, 23, 24]. Interestingly, a global survey on low-vision service provision found both cost and distance to the nearest LVR service to be more common barriers in developing countries [22]. Additional challenges to accessing LVR in developed countries include lack of awareness, lack of referrals, and poor communication between patients and providers [22].

The findings of this study provide additional insights into the holistic model for LVR proposed by Luu et al. which included physical, functional, psychological, and social factors as the four major areas for assessment. Most low-vision patients are elderly, and many have additional medical comorbidities that limit their physical participation in LVR services. Poor physical health is a common barrier identified by both providers and patients in accessing LVR. Accordingly, physical health should be addressed by the provider when making recommendations.

Previous research has shown improved referral rates for providers with integrated ophthalmology and low-vision services [28]. Our study was performed in an integrated practice, where the majority (78%) of ECPs were aware of low-vision services. However, many ECPs did not refer eligible patients to LVR when barriers such as poor health, declining cognitive function, old age, lack of social support, or perceived low likelihood of keeping an appointment were factors. In these scenarios, barriers to LVR services directly inhibited needed care.

Of the modifiable barriers identified by the patients, the denial of need of LVR services can be addressed by increasing patient understanding of low vision and educating patients on available low-vision aids and rehabilitation services. Counseling patients in the presence of a family member, especially in patients with cognitive impairment, will help patients and families understand the benefits of LVR and may further motivate them to utilize the services. Sarika et al. proposed establishing a counseling chamber, where explanation of the ocular condition, visual prognosis, and available LVR services can be explained to low-vision patients [21]. A multidisciplinary approach with the primary care providers, occupational therapists, social workers, and counselors will further assist in providing better LVR access to patients with mental health issues and lack of social support.

## 4. Conclusions

To our knowledge, this is the first study investigating barriers to low-vision rehabilitation services in both patients and referral providers in a hospital-based integrated practice. Our study elucidated factors such as patient's overall health, mental health or cognitive decline, social support, and transportation issues as critical barriers for LVR services. Knowledge of the common barriers perceived by patients and providers allows these obstacles to be addressed, whether by establishing new protocols and new programs or raising awareness. Furthermore, some of the main barriers identified are indirect indications for LVR referral. Decline in vision is associated in some studies with increased prevalence of types of dementia [29, 30]. Because these two diseases heavily overlap, earlier interventions are more likely to be of benefit through increased patient understanding and compliance. Therefore, ECPs should strongly consider early referral for patients with cognitive decline or mental health concerns. Similarly, low vision should be especially addressed in patients with multiple comorbidities [31]. Concurrently, a multidisciplinary approach educating and helping the patient access services would overcome many of the common patient-identified barriers.

## Figures and Tables

**Table 1:** Etiology of low vision for eligible patients (*n* = 190).

Etiology	% of eligible patient populations
ARMD	35
Diabetic retinopathy	16
Glaucoma	16
Optic neuropathy	5
Retinal detachment	3
Chronic uveitis	2
Other	21

ARMD = age-related macular degeneration.

## Data Availability

The patient data used to support the findings of this study are restricted by the University of Texas Medical Branch Institutional Review Board (approval number 20-0112) in order to protect patient privacy. Data are available from Praveena Gupta, PhD, OD, FAAO (prgupta@utmb.edu), for researchers who meet the criteria for access to confidential data.
